# Genetic mapping in collaborative cross mouse strains identifies loci that affect initial sensitivity to cocaine

**DOI:** 10.1007/s00213-025-06901-z

**Published:** 2025-10-27

**Authors:** Sarah A. Schoenrock, Christiann H. Gaines, Padam Kumar, Saad Khan, Joseph Farrington, Martin T. Ferris, Fernando Pardo-Manuel de Villena, William Valdar, Jason A. Bubier, Lisa M. Tarantino

**Affiliations:** 1 Department of Genetics, School of Medicine, University of North Carolina, Chapel Hill, NC, USA; 2 The Jackson Laboratory, Bar Harbor, ME, USA; 3 Division of Pharmacotherapy and Experimental Therapeutics, Eshelman School of Pharmacy, University of North Carolina, Chapel Hill, NC, USA

**Keywords:** QTL, Locomotor, Acute, Addiction, Gene discovery, RI strains

## Abstract

**Rationale:**

Cocaine use disorder (CUD) is a significant health concern that has devastating impacts on affected individuals and society. There are currently no approved therapies to treat CUD due, in part, to significant gaps in our knowledge about the underlying factors that increase risk. Individual genetic differences contribute to CUD risk. Identifying specific genetic mechanisms that increase risk could reveal novel targets for treatment and prevention.

**Objectives:**

We identified two Collaborative Cross (CC) strains, CC004/TauUncJ (CC004) and CC041/TauUncJ (CC041), that differ significantly for locomotor response and self-administration of cocaine. In the current study, we used mapping crosses generated from both strains to identify genetic loci that are associated with behavioral responses to cocaine.

**Results:**

We bred mice from the low (CC041) and high (CC004) responding strains to C57BL/6NJ mice to produce two F2 populations and identify genetic loci that influence locomotor response to cocaine. We identified three significant loci on chromosomes 7, 11 and 14 in the CC041 F2 mapping cross that collectively explain 14% of the phenotypic variance for locomotor response to cocaine. Bioinformatic analyses identified multiple genes on chromosomes 7 and 11 that are genetically plausible, have functional relevance and are suitable for further exploration.

**Conclusions:**

Genetically defined and phenotypically divergent mouse strains are a useful tool for identifying candidate genes that influence behavioral responses to psychostimulants. Functional and mechanistic analyses of these genes could provide insights into biological processes that increase risk for CUD.

## Introduction

Approximately 2.2 million individuals in the United States use cocaine regularly and 1.5 million meet the diagnostic criteria for Cocaine Use Disorder (CUD) (Substance Abuse and Mental Health Services Administration 2017, 2020). The rate of cocaine use has increased in recent years and cocaine-related overdose deaths have also increased (John and Wu 2017; Cano et al. 2020; European Monitoring Centre for Drugs and Drug Addiction 2024). Despite the significant personal, societal and financial burdens imposed by CUD, there are currently no FDA-approved treatments. Addressing gaps in our knowledge about underlying mechanisms that lead to CUD would aid in identifying novel targets and developing effective treatment and preventative strategies.

Among addictive substances, cocaine has one of the highest risks of dependence (Goldstein and Kalant 1990). Twin studies indicate that the heritability (*h*^*2*^) of cocaine dependence ranges from 0.4—0.7 indicating that genetics play a significant role (Goldman et al. 2005; Ducci and Goldman 2012). A human GWAS for cocaine dependence identified a significant association with a single nucleotide polymorphism (SNP) in the FAM53B (family with sequence similarity 53, member B) gene on chromosome (Chr) 10 (Gelernter et al. 2014) in a region that was identified by linkage analysis in a previous study (Gelernter et al. 2005). Despite a few successful studies, GWAS for CUD have been limited by insufficient sample sizes that reduce power to detect the likely numerous genetic variants that drive cocaine use and risk for CUD (Goldman et al. 2005).

Genome-wide mapping approaches have been employed in mice and overcome some of the obstacles present in humans. The use of experimental mice allows for control over drug exposures, minimizes environmental variability and provides access to brain tissue for mechanistic studies. Moreover, gene by drug interactions can be assessed by studying the effects of drug exposure on different genetic backgrounds. Genetic mapping in mice has identified numerous quantitative trait loci (QTL) for addiction-relevant behaviors (Phillips et al. 1998; Dickson et al. 2016; Bubier et al. 2020; Vadasz and Gyetvai 2020; Bagley et al. 2022). In rodents, acute psychomotor response to cocaine is commonly used as a measure of initial sensitivity to the drug. Human studies have shown that initial response to a drug predicts future use and abuse (de Wit and Phillips 2012). Preclinical rodent studies also provide evidence that initial locomotor response to psychostimulants predicts contingent drug exposure in self-administration paradigms (Deminiere et al. 1989; Piazza et al. 1989; de Wit and Phillips 2012). QTL studies for initial cocaine sensitivity have been conducted using standard recombinant inbred (RI) strains (Tolliver et al. 1994; Miner and Marley 1995; Phillips et al. 1998; Jones et al. 1999; Boyle and Gill 2001, 2009; Gill and Boyle 2003, 2008) or C57BL/6 substrains (Kumar et al. 2013) and have identified numerous genomic regions. To date, however, only one genetic variant in the *Cyfip2* gene has been identified and validated for initial locomotor sensitivity (Kumar et al. 2013). Identification of genes that influence initial locomotor sensitivity to cocaine will inform mechanistic studies that will further our understanding of specific factors that might drive risk for CUD.

In the present study, we utilized a new resource, the Collaborative Cross (CC). The CC was created by crossing eight inbred mouse strains including five classical strains – A/J (A), C57BL/6 J (B6J), 129S1/SvImJ (129), NOD/ShiLtJ (NOD), NZO/HlLt (NZO) and three wild-derived strains – WSB/EiJ (WSB), CAST/EiJ (CAST), PWK/PhJ (PWK). The CC is unique in its genetic diversity as the eight inbred founders represent the three subspecies of *Mus musculus* (Churchill et al. 2004; Srivastava et al. 2017). The extensive genetic diversity allows for novel combinations of alleles and the observation of expanded phenotypic diversity in comparison to traditional inbred strains and RI panels (Rasmussen et al. 2014; Graham et al. 2015; Gralinski et al. 2015; Mosedale et al. 2017). Moreover, the availability of an expanded set of genetic and genomic tools developed to support CC studies allows for systems genetic analysis and the ability to move more quickly from QTL to candidate gene analyses (Ball et al. 2024).

In a previous study, we identified two CC strains that exhibit low (CC041/TauUnc; CC041) or high (CC004/TauUnc; CC004) initial locomotor response to cocaine. Low responding CC041 mice are also unable to acquire intravenous cocaine self-administration in comparison with CC004 mice which acquire at a normal rate (Schoenrock et al. 2020) suggesting that both the activating and reinforcing effects of cocaine differ in these two strains. We crossed both CC strains to C57BL/6NJ (B6N) to generate two separate F2 mapping populations. The B6N strain was chosen because it is a substrain of C57BL/6 J (B6J), one of the eight CC founder strains. Thus, B6N mice are genetically similar to B6J mice, and as such, do not introduce an entirely new genetic background. However, the B6N substrain still carries enough SNP markers that distinguish it from B6J for genome-wide genetic mapping. QTL mapping using the CC041 × B6N F2 identified significant QTLs on Chrs 7, 11 and 14. A similar mapping strategy in the CC004 × B6N F2 yielded only one suggestive locus. We used extensive in silico bioinformatic analyses to select candidate genes at the CC041 Chr 7 and 11 QTL peaks that have a high likelihood of being causal. These genes can be characterized in future studies to test their role in addiction-relevant behaviors in preclinical models and in substance use disorders in humans.

## Methods and materials

### Animals

Mice from the CC004/TauUncJ (CC004) strain were purchased from the Jackson Laboratory in November 2015. Mice from the CC041/TauUnc (CC041) strain were purchased from the Systems Genetics Core Facility at the University of North Carolina (UNC; http://csbio.unc.edu/CCstatus/index.py) in January 2016. CC004 and CC041 mice were rec iprocally bred to C57BL/6NJ (B6N; RRID:MGI:3056279) mice purchased from The Jackson Laboratory (Bar Harbor, ME) to generate CCxB6N and B6NxCC F1s (denoted as BC F1 and CB F1). F1s were crossed in all combinations (CCxB6N × CCxB6N, CCxB6N × B6NxCC, B6NxCC × CCxB6N, B6NxCC × B6NxCC denoted as CBCB, CBBC, BCCB and BCBC) to generate each of the two (CC004 and CC041) F2 populations.

Animals were housed in a specific pathogen-free facility on a 12-h light/dark cycle with lights on at 7:00 A.M. Food and water were available ad libitum throughout the experiment. Breeder diet was Harlan Teklad 2919 and post-weaning diet was Harlan Teklad 2920 (Envigo, Frederick, MD, USA).

### Drugs

Cocaine HCl (Sigma-Aldrich, St. Louis, MO) was dissolved in 0.9% saline. A 20 mg/kg dose was injected via the intraperitoneal route at a volume of 0.01 ml/g.

### Phenotyping for initial locomotor sensitivity to cocaine

Founder strain (CC004 and CC041), F1 and F2 mice were tested for initial locomotor response to cocaine using a three-day open field (OF) assay. Total numbers of mice tested for each group and the average age of each strain, cross and generation are shown in Table 1. On Days 1 and 2, mice were administered saline at a volume of 0.01 ml/g via the intraperitoneal (i.p.) route and immediately placed into a 43.2 × 43.2 × 33 cm open field arena (ENV-515-16, Med Associates, St. Albans, VT, USA). Mice were tracked for the entire session by infrared detectors that surrounded the arena at 2.54 cm intervals on the x, y, and z axes. After 30 min, mice were removed from the OF and returned to the home cage. On Day 3, mice were given an i.p. injection of 20 mg/kg cocaine at a volume of 0.01 ml/g and placed in the OF for 30 min. On each day, distance moved (in centimeters) was recorded using behavioral software provided by the OF manufacturer (Med Associates; RRID:SCR_014296). Total distance moved during the entire 30-min assay was used as a dependent variable.

### Statistical analyses

All behavioral data were normalized using a rank-based inverse normal transformation (RINT). Transformed values were distributed around original group means and standard deviations. Prior to statistical analyses, equality of variances was tested using Levene’s test in SPSS (v28 for Mac OS, IBM Corp; RRID:SCR_016479). An ANOVA of the effects of day, strain and sex on normalized distance in the OF was performed for parental and F1 strains and for F2 mice using SPSS. A separate ANOVA tested the effects of strain and sex on normalized locomotor response to cocaine on Day 3 minus baseline activity on Day 2 (Day 3 – Day 2). For both analyses, mice from various reciprocal crosses in the F1 and F2 were analyzed as separate groups to identify parent of origin effects that might impact mapping analyses. Post hoc Tukey’s *HSD* was used to follow-up on significant main effects of strain and day. Post hoc t-tests were used to assess sex effects. Graphs were generated using GraphPad Prism 10 for Mac OS (GraphPad Software, LLC; RRID:SCR_002798). Power analyses for both F2 crosses were conducted using G*Power (Faul et al. 2007).

### Genotyping

Genotyping was performed for 448 CC041 × B6N F2 mice and 151 CC004 × B6N F2 mice. Since most of the power for genetic mapping is provided by the animals at the extreme ends of the phenotypic distribution, we initially genotyped only the phenotypic extremes in both mapping populations. Selective genotyping as a mapping strategy works well when the samples are densely genotyped (Sen et al. 2005) as they are in both of our F2 populations. In the CC041 × B6 F2 population, we detected a strong signal in the mapping analyses of extreme individuals, and we completed genotyping the entire F2 population. For the CC004 × B6N F2 population, initial mapping in 151 genotyped animals (out of 309 phenotyped animals) yielded no significant or suggestive QTLs. Therefore, we determined that adding additional animals to the analyses might not be productive and we decided to devote resources to completing genotyping in the CC041 F2 mice. Four CC041 × B6N F2 mice were excluded from the genetic mapping analyses due to technical issues during genotyping resulting in a final sample size of 444 mice.

Mice were euthanized by CO_2_ inhalation immediately following testing on Day 3 and DNA was extracted from tail tissue of CC parents, F1 breeders, representative B6N samples and the F2 population using the DNeasy Blood & Tissue kit (Qiagen). Genotypes for the CC041 × B6N F2s were determined using the Mouse Universal Genotyping Array (MUGA) that consists of 7851 SNP markers on an Illumina Infinium platform that are distributed throughout the genome (average spacing of 325 kb) and were chosen to be maximally informative for the eight founder strains of the CC (Morgan et al. 2015). Genotypes for the CC004 × B6N F2s were determined using the fourth iteration of the Mouse Universal Genotyping Array (MiniMUGA). The MiniMUGA contains a total of 10,821 genomic probes that survey the most informative SNPs from 120 classical inbred lines of mice (Sigmon et al. 2020; Blanchard et al. 2024). Nucleotide genotypes were processed and converted to haplotype calls (i.e. B6N, CC, or HET) for use in QTL mapping using the *argyle* package (version 0.2.0) in R Studio (Morgan 2015). A series of genotype checks were performed, and markers were eliminated for any of the following reasons: markers were not informative between the CC and B6N, did not meet the Chi-square distribution of expected genotypes for an intercross population or had greater than 40 missing calls. In the CC041 × B6N F2 population, this strategy resulted in a final marker set that included 2701 markers with an approximate average spacing of 1 megabase (Mb) and maximum gap of 14.7 Mb on Chr 10. For the CC004 × B6N F2 population, the final marker set included 2394 markers with an average spacing of approximately 2 Mb and a maximum gap of 60 Mb on Chr 15. Both marker sets had more than ample coverage for mapping in an F2 population.

### QTL mapping

All F2 data were transformed by RINT using the GenABEL package in R (F2) or SPSS (F0, F1). Data were distributed around the original group means and standard deviations. QTL mapping was performed using the *qtl* package (version 1.40–8;Broman et al. 2003; Broman 2014) in R Studio (version 1.0.136). Single scan QTL analyses using the *scanone* function were performed for transformed total distance data for Day 1, Day 2 and Day 3 and D3-D2 distance. Sex and F2 breeding cross direction (BCBC, CBBC….etc.) were included as covariates. A Haley-Knott regression approximation model of interval mapping was used based on the density of the genotyping array and the amount of recombination present in the F2 intercross. Genome-wide significant thresholds for logarithm of the odds (LOD) scores (measure of genotype to phenotype association) were determined using 1000 permutations. For each QTL peak identified, the 1.5 LOD support interval was used as it can be translated to having a 95% likelihood for containing the casual variant (Dupuis and Siegmund 1999). The MUGA or MiniMUGA markers closest to the outer limits of the 1.5 LOD interval were identified and used as the megabase (Mb) locations flanking the region (mm10, GRCm38). Genotype data provided in Srivastava et al. (2017) was used to determine the CC parental strain haplotype in the 1.5 LOD intervals for each QTL. The amount of variance explained by each of the QTL was estimated with the *fitqtl* function in *R/qtl* using an equation assuming an additive model, (e.g. *y* = *QTL 1* + *QTL 2* + *QTL 3*).

Two QTL analysis using the *scantwo* function in *R/qtl* was performed for the significant QTLs identified for each of the measures with sex and cross direction as covariates. Both full (LOD_*fv1*_) and additive models (LOD_*av1*_), allowing for the possibility of epistasis or not, respectively, were fit by comparing pairs of loci on two chromosomes to the single-QTL model. LOD_*i*_ indicates evidence for an interaction of the two loci by comparing the fit of the two models (LOD_*fv1*_—LOD_*av1*_). Thresholds of 6, 4, 3 for LOD_*fv1*_, _*i*_, _*av1*_ were used to determine significant pairs of QTLs (Broman and Sen 2009). Effect plots at each QTL peak and for significantly interacting markers were generated using the *effectplot* function.

### Prioritizing candidate genes in CC041 QTL regions

We prioritized potential candidate genes in the QTL intervals using a multi-step process. We queried the Genome-MUSter website (Ball et al. 2024) (https://mpd.jax.org/genotypes) to identify SNPs in coding regions that varied between CC041 and B6N in the Chr 7 (27.5–37.9 Mb) and Chr 11 (12.6–37.7 Mb) QTL intervals. We prioritized deleterious polymorphisms such as STOP loss or gain mutations as well as missense or non-synonymous amino acid mutations. In order to determine if amino acid changes were predicted to be tolerated or disruptive, the SNP ids were tested using SIFT (https://sift.bii.a-star.edu.sg/; RRID:SCR_012813)(Sim et al. 2012). We also selected SNPs that reside in evolutionarily conserved residues by performing multi-species protein sequence alignment, retrieving protein amino acid sequences from UniProt (http://www.uniprot.org; RRID:SCR_002380)(UniProt 2023) and performing multiple sequence alignments using CLUSTAL Omega (https://www.ebi.ac.uk/Tools/msa/clustalo/ RRID:SCR_001591)(Sievers et al. 2011).

## Results

### Locomotor response to cocaine in the CC041 population

**Fig. 1:**
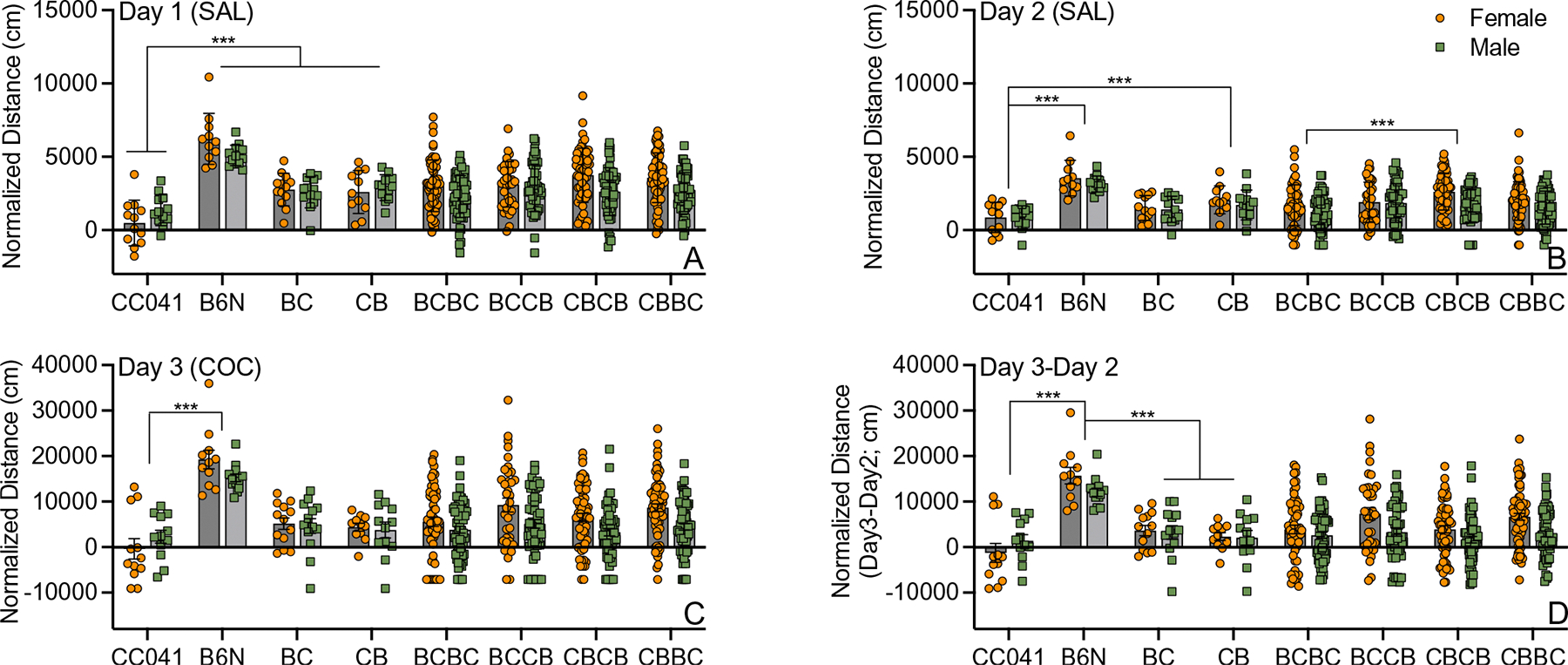
Locomotor activity in response to saline on Day 1 (**A**) and Day 2 (**B**), after cocaine exposure on Day 3 (**C**) and Day 3 minus Day 2 (**D**) in CC041 and B6N parent strains, reciprocal F1 mice and all F2 cross types. Data are normalized using the rank-based inverse normal transformation and distributed around the strain mean and standard deviation. B6N mice are significantly more active than CC041 mice on all three days (**A-C;** all *p* < 0.001) and significantly more active than both F1 crosses in response to cocaine (**D;***p* < 0.001). BC = B6N × CC041 F1, CB = CC041 × B6N F1, BCBC = BC F1 × BC F1, BCCB = BC F1 × CB F1, CBCB = CB F1 × CB F1 and CBBC = CB F1 × BC F1. Strain background of dam is listed first

The number of mice tested in each generation in both mapping populations is provided in Table 1. We performed an analysis of variance (ANOVA) in the two parental strains (CC041 and B6N) and F1 offspring (BC and CB) to determine the effects of day, strain and sex on transformed locomotor activity (Fig. 1A–C). We observed a significant main effect of strain (F_(2,267)_ = 76.5; *p* = 9.3 × 10^−36^; *η*^2^p = 0.46) and day (F_(2,267)_ = 59.6; *p* = 59.6 × 10^−22^; *η*^2^p = 0.31) but no sex effect (F_(1,267)_ = 0.57; *p* = 0.452). Locomotor activity was significantly lower on day 2 compared to day 1 (*p* = 0.049) and significantly higher on day 3 compared to both day 2 (*p* = 5.1 × 10^−9^) and day 1 (*p* = 5.1 × 10^−9^). B6N mice were significantly more active than CC041 and F1 mice (all *p* < 0.001) regardless of day. However, we also observed a significant strain by day interaction (F_(6,267)_ = 24.8; *p* = 2.7 × 10^−23^; *η*^2^*p* = 0.36). CC041 mice were significantly less active than B6N mice and both BC and CB F1 mice on Day 1 (*p* < 0.001). On Day 2, distance traveled by CC041 mice still differed from CB (*p* < 0.001) but not BC mice (*p* = 0.103). By Day 3, locomotor behavior did not differ between CC041 mice or either F1 cross but still differed significantly from B6N mice (*p* < 0.001). No parent of origin effect was observed in the F1 as BC F1 mice did not differ significantly from CB F1 mice on any of the 3 days (all *p* > 0.05).

An ANOVA in the CC041 F2 population yielded significant day (F_(2,1320)_ = 109.6; *p* = 9.6 × 10^−45^; *η*^2^*p* = 0.14), cross (F_(3,1320)_ = 4.2; *p* = 0.006; *η*^2^*p* = 0.01) and sex effects (F_(1,1320)_ = 44.9; *p* = 3.0 × 10^−11^; *η*^2^*p* = 0.03) as well as day by cross (F_(6,1320)_ = 2.9; *p* = 0.01; *η*^2^*p* = 0.01) and day by sex (F_(2,1320)_ = 15.8; *p* = 0.02;; *η*^2^*p* = 0.02) interaction effects (Figs. 1A–C). The pattern of locomotor behavior in the F2 mice across the three days of testing was the same as that observed in the parental strains and F1 mice. F2 mice were significantly less active on Day 2 compared to Day 1 and more active on Day 3 compared to either Day 1 or Day 2 (all *p* < 0.001). BCBC mice (N = 120) were significantly less active than BCCB (N = 87; *p* = 0.02) and CBBC mice (N = 129; *p* = 0.02). No other significant differences were observed among the different F2 cross types. Overall, the CC041 × B6N F2 population showed a wide range of responses, although a large proportion of mice exhibited locomotor activity at the lower end of the phenotypic distribution appearing more like the CC041 parent, which is consistent with our observations in the F1 and parental strains (Fig. 1). The day by cross interaction reflected significantly lower activity in BCBC F2 mice compared to CBCB F2 mice on Day 2 (*p* < 0.001). No significant differences in locomotor activity between the reciprocal F2 crosses were observed on any other day. Female mice of all F2 cross types had significantly higher locomotor activity than male mice (all *p* < 0.01) – a difference that persisted across all days of testing in response to both saline and cocaine.

ANOVA of initial locomotor response to cocaine on Day 3 minus Day 2 in the parental strains and F1 mice yielded a significant effect of cross (F_(3,89)_ = 35.5; *p* = 3.4 × 10^−15^; *η*^2^*p* = 0.54) but not sex (F_(1,89)_ = 0.17; *p* = 0.68) (Fig. 1D). B6N mice were significantly different from BC and CB F1 mice and CC041 mice (all *p* < 0.001). In the F2 population, we observed a significant effect of cross (F_(3,440)_ = 4.1; *p* = 0.007; *η*^2^*p* = 0.03), but post hoc analyses identified no significant cross differences. We also observed a significant sex effect (F_(1,440)_ = 23.6; *p* = 1.7 × 10^−6^; *η*^2^*p* = 0.05): female mice were significantly more active than male mice.

Based on the size of our CC041 F2 mapping population (N = 444), we had a 99% probability of detecting a QTL controlling 5% of the variance.

### Locomotor response to cocaine in the CC004 population

**Fig. 2:**
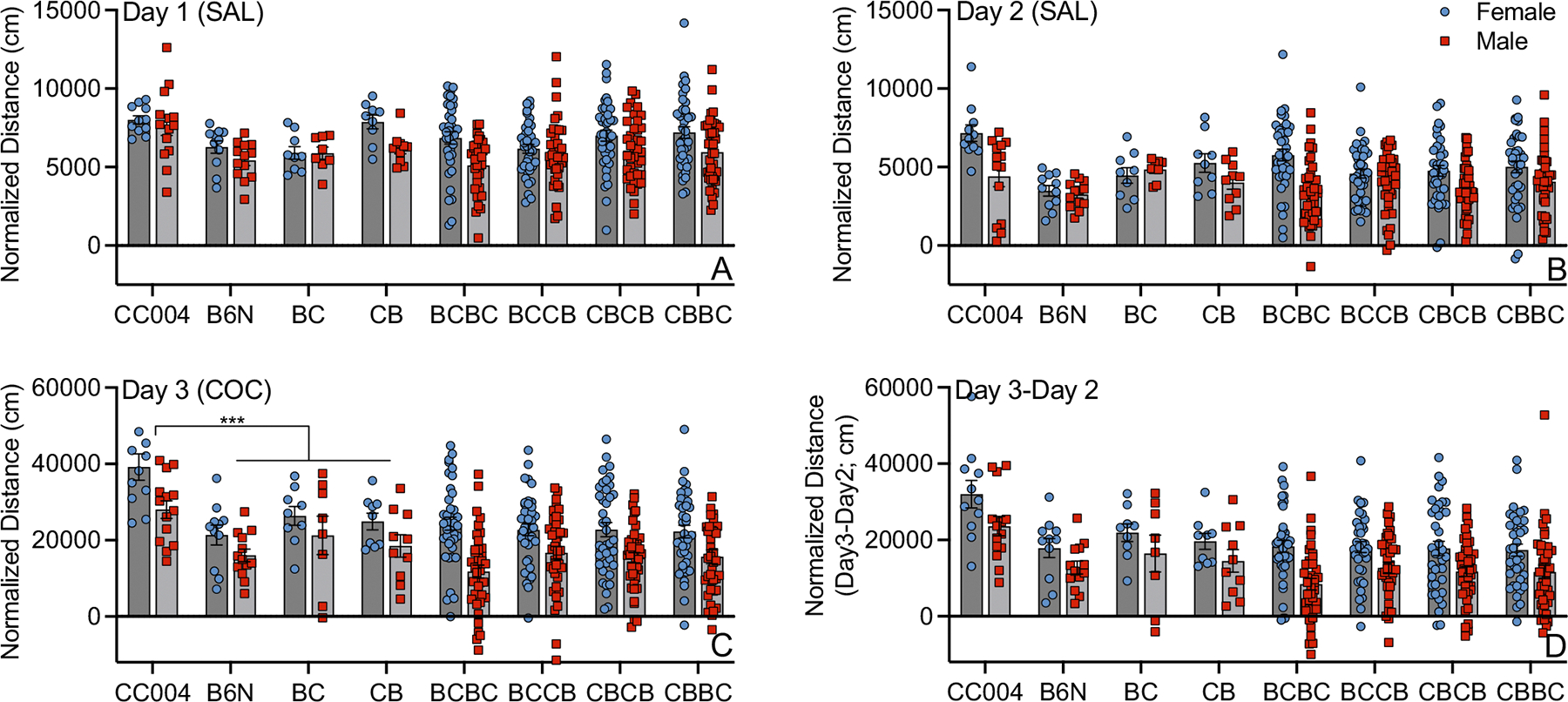
Locomotor activity in response to saline on Day 1 **(A)** and Day 2 **(B)**, after cocaine exposure on Day 3 (**C**) and Day 3 minus Day 2 (**D**) in CC004 and B6N parent strains, reciprocal F1 mice and all F2 cross types. Data are normalized using the rank-based inverse normal transformation and distributed around the strain mean and standard deviation. CC004 mice are significantly more active than B6N, BC and CB mice in response to cocaine on Day 3 (**C**; all *p* < 0.001). BC = B6N × CC004 F1, CB = CC004 × B6N F1, BCBC = BC F1 × BC F1, BCCB = BC F1 × CB F1, CBCB = CB F1 × CB F1 and CBBC = CB F1 × BC F1. Strain background of dam is listed first

We performed an ANOVA on CC004 and B6N parental strains and BC and CB F1 offspring to assess the effects of strain (cross), sex and day on locomotor activity (Fig. 2A–C). Strains differed significantly (F_(3,231)_ = 18.4; *p* = 1.0 × 10^−12^; *η*^2^*p* = 0.19) with CC004 mice exhibiting significantly higher locomotor activity across all days than B6N, BC F1 and CB F1 mice (all *p* < 0.001). However, a significant strain by day interaction (F_(6,231)_ = 8.6; *p* = 1.7 × 10^−8^; *η*^2^*p* = 0.18) indicated that locomotor activity in CC004 mice did not differ from CB mice on Day 1 or from BC and CB mice on Day 2. On Day 3, CC004 mice were significantly more active than all other strains (all *p* < 0.02). All strains exhibited significantly higher locomotor activity on Day 3 compared to Day 1 and Day 2 (F_(2,231)_ = 335.5; *p* = 4.7 × 10^−69^; *η*^2^*p* = 0.74). Females were more active than male mice (F_(1,231)_ = 17.7; *p* = 3.7 × 10^−5^; *η*^2^*p* = 0.07). We also observed a significant day by sex interaction (F_(2,231)_ = 8.8; *p* = 2.0 × 10^−4^; *η*^2^*p* = 0.06). Females exhibited higher locomotor activity than males on all three days, but the difference was only significant on Days 2 and 3 (all *p* < 0.01).

An ANOVA in the CC004 F2 population yielded significant main effects of day (F_(2,900)_ = 492.9; *p* = 2.7 × 10^−145^; *η*^2^*p* = 0.52) and sex (F_(1,900)_ = 71.0; *p* = 1.4 × 10^−16^; *η*^2^*p* = 0.07) on locomotor activity (Fig. 2D). Female mice were more active in the open field than male mice. Locomotor activity in all groups was significantly higher after cocaine exposure on Day 3 than after saline on Day 1 or Day 2 (all *p* < 0.001). Locomotor activity was also significantly lower on Day 2 than Day 1 (*p* < 0.001) indicating that mice habituated to the open field after the first exposure. We also observed a day by sex interaction effect (F_(2,900)_ = 31.9; *p* = 4.2 × 10^−14^; *η*^2^*p* = 0.07). Females were significantly higher than males on all three days, but the difference was greater after cocaine exposure on Day 3 compared to the saline treatment days. We observed no significant effect of cross (F_(3,900)_ = 0.31; *p* = 0.82) indicating that parent of origin had no effect on the behavior.

An ANOVA of acute locomotor response to cocaine (Day 3 minus Day 2) in the parental strains and F1 crosses yielded significant effects of strain (F_(3,77)_ = 8.6; *p* = 5.3 × 10^−5^; *η*^2^*p* = 0.25) and sex (F_(1,77)_ = 8.8; *p* = 0.004; *η*^2^*p* = 0.10). CC004 mice had a higher acute response to cocaine in comparison to B6N (*p* = 8.4 × 10^−5^), BC (*p* = 0.036) and CB (*p* = 0.002). B6N and BC and CB F1 mice did not differ in their response to cocaine. In the F2 population, we observed significant sex effects (F_(1,301)_ = 37.6; *p* = 2.8 × 10^−9^; *η*^2^*p* = 0.11). We observed no cross effects (F_(3,301)_ = 0.59; *p* = 0.622) indicating that parent of origin did not influence initial locomotor sensitivity to cocaine in the CC004 mapping population.

Based on the size of the genotyped CC004 F2 mapping population, we had a 68% probability of detecting a QTL accounting for 5% of the variance and a 94% probability of detecting a QTL accounting for 10% of the variance.

### QTL on Chr 7, 11 and 14 for cocaine-induced locomotor activation in CC041 × B6N population

**Fig. 3:**
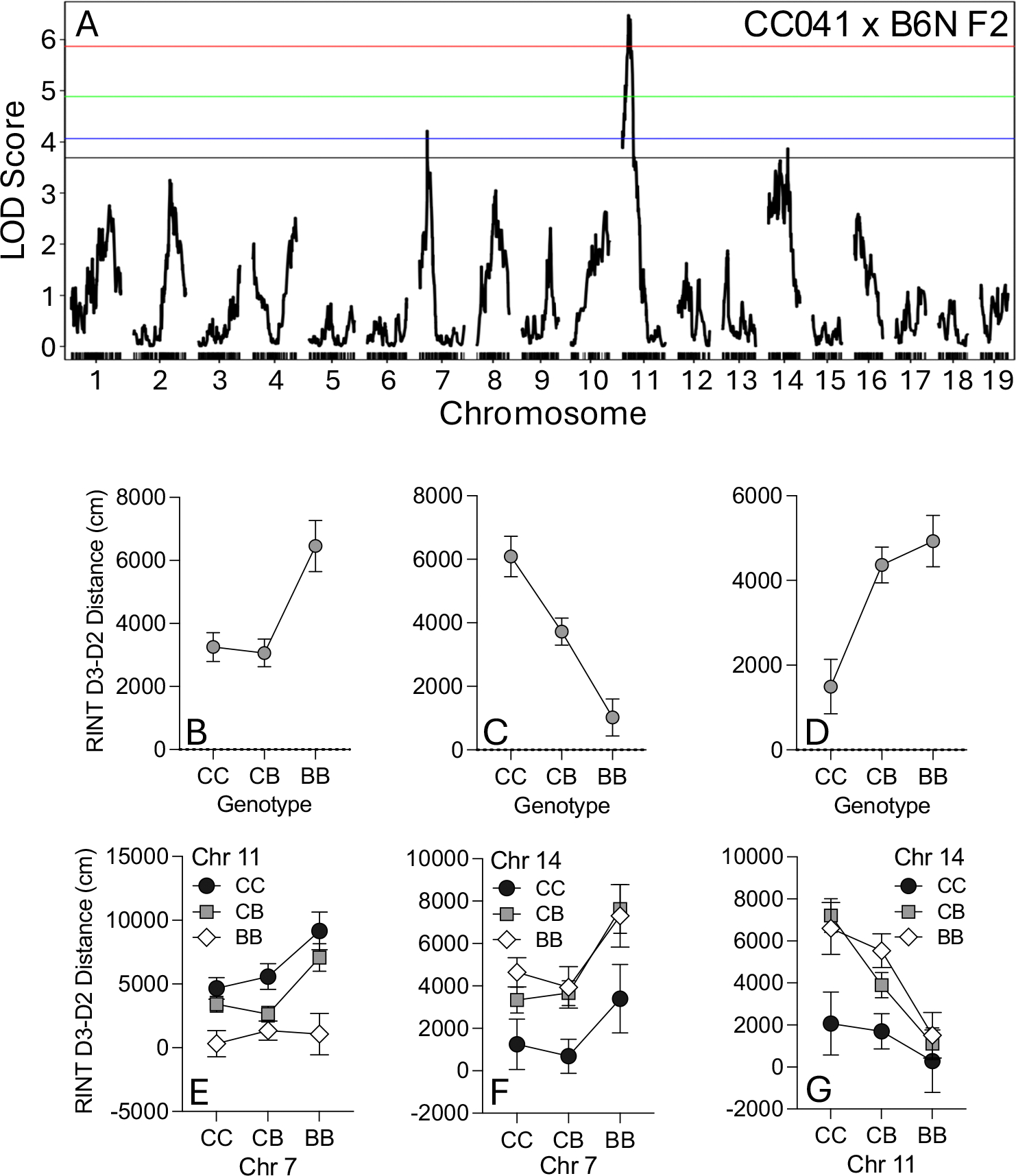
(**A**) Single scan QTL for initial cocaine response measured as Day 3 minus Day 2. Genome-wide significant LOD thresholds based on 1000 permutations for *p* = 0.001 (red line), *p* = 0.01 (green line), *p* = 0.05 (blue line), and suggestive at *p* = 0.1 (black line). QTL effect plots for Chr 7 (**B**), 11 (**C**) and 14 (**D**) and two-scan interaction plots for Chr 7 vs Chr 11 (**E**), Chr 7 vs Chr 14 (**F**) and Chr 11 vs Chr 14 (**G**). CC = CC041/CC041 homozygote, CB = CC041/B6N heterozygote and BB = B6N/B6N homozygote. Error bars are SEM

We conducted genetic mapping in our CC041 × B6N population and identified 3 significant QTL for locomotor response to cocaine (Day3-Day2 distance) on Chr 7, 11 and 14 (Fig. 3A, Suppl Fig. 1A). QTLs that passed the *p* < 0.1 genome-wide LOD threshold based on 1000 permutations are shown in Table 2. The QTL on Chr 7 had a peak at 29.7 Mb (LOD = 4.22, *p* = 0.034) and accounted for 4.3% of the variance. The effect plot at this peak showed that both the CC041/CC041 and HET genotypes were driving the low cocaine response (Fig. 3B), consistent with our initial observations of dominance of the CC041 background in the parental and F1 populations. The LOD interval (27.5–37.9 Mb) of the Chr 7 peak is 10.4 Mb and contains 203 protein-coding genes. CC041 has a NOD haplotype that transitions to NZO at approximately 36.05 Mb in this interval (Table 2). The QTL on Chr 11 had a peak at 23.6 Mb (LOD = 6.48, *p* = 0.001) and accounted for 6.1% of the variance. The effect plot at this peak showed that the B6N/B6N genotype was driving the low cocaine response (Fig. 3C) indicating a transgressive QTL. The LOD interval (12.6–37.7 Mb) of the Chr 11 peak is 25.1 Mb and contains 106 protein-coding genes. CC041 has a NOD haplotype that transitions to a WSB haplotype at approximately 36.8 Mb. The Chr 14 QTL had a peak at 79.7 Mb (LOD = 3.88, *p* = 0.074) that accounted for 4%of the variance. The effect plot at this peak showed that the CC041/CC041 genotype was driving the low cocaine response (Fig. 3D). Due to the presence of multiple peaks, the Chr 14 LOD interval (9.2–97.5) spanned most of the chromosome (88.4 Mb) and we did not perform follow-up analyses of potential candidate genes in this region.

Single QTL analysis of Day 1, Day 2 and Day 3 distance revealed several overlapping regions (Table 2, Suppl Fig. 1B–D, Suppl Fig. 2). A peak on Chr 11 was identified at a similar location for all 3 days and in every case, the B6N/B6N genotype was associated with the low response. Overlapping QTL regions on Chr 7 were identified for Day 2 and Day 3 distance and in both cases, the CC041/CC041 homozygous and CC041/B6N heterozygous genotypes were associated with low response. Overlapping QTL regions were also identified on Chr 14 for Day 1 and Day 3 locomotor activity and in all cases only the CC041/CC041 genotypes showed a low response. Two separate QTL peaks on Chr 6 were specific to non-cocaine (saline) exposures on Day 1 (peak at 97.0 Mb) and Day 2 (at 37.0 Mb). Although the peaks were in different locations, overlapping 1.5 LOD support intervals might indicate the same causal gene or genes. For both Chr 6 QTL, the CC041/CC041 genotype was associated with low locomotor response.

Collectively, the single scan QTL analysis in the low responding population indicates that the QTL identified are associated with locomotor phenotypes on multiple days in the 3-day OF test, a finding that could be due to the significant correlation among the phenotypes (Suppl Table 1). Pearson correlation showed that Day 1, Day 2, Day 3 and Day 3-Day 2 distance were all significantly correlated (r(6) = 0.31–0.99; *p* < 1 × 10^−15^). The strongest correlation was observed between Day 3 distance and D3-D2 distance (r(6) = 0.99; *p* < 1 × 10^−15^).

### Two-locus analysis shows additive effect of QTLs for low COC response in CC041 × B6N population

Complex traits, such as initial drug sensitivity, are likely driven by the action of multiple genetic loci, some of which may act in concert to affect the phenotype. Therefore, we performed a two-locus analysis to determine if the QTLs identified in the single scan are interacting and if so, in what manner (i.e. additive or epistatic). For each phenotype, only the chromosomes with significant QTLs in the single scan were analyzed. Table 3 shows all loci that met the threshold in either a full model (allows for possibility of epistasis; LOD_*fv1*_ ≥ 6.0) or additive model (assumed no epistasis; LOD_*av1*_ ≥ 3.0).

For Day3-Day2, we found evidence for an interaction among a pair of QTL at the same locations identified in the single scan analyses on Chrs 7 and 11. The presence of a homozygous B6N genotype on Chr 11 results in low response to cocaine regardless of the genotype on Chr 7 (Fig. 3E). There was also evidence for an interaction between QTL on Chrs 7 and 14. Although the Chr 7 QTL effect is preserved across genotypes, CC041 homozygosity at the Chr 14 locus results in reduced locomotor response to cocaine across all three Chr 11 genotypes. There was also evidence for interaction between a pair of QTL on Chrs 11 and 14 (Fig. 3F). CC041 homozygosity at the Chr 14 locus represses the allelic effects of the Chr 11 QTL on locomotor response to cocaine (Fig. 3G). We note, however, that none of these potential interactions met the level of significance.

### QTL in CC004 × B6N population

A genome-wide scan in the CC004 × B6N F2 cross identified a suggestive QTL peak at the distal end of Chr 11 at 118 Mb or (LOD = 3.76). (Table 2). No other QTL were identified in this cross.

### Prioritization of candidate genes within the Chr 7 and Chr 11 QTL regions identified in CC041 × B6N population

**Fig. 4:**
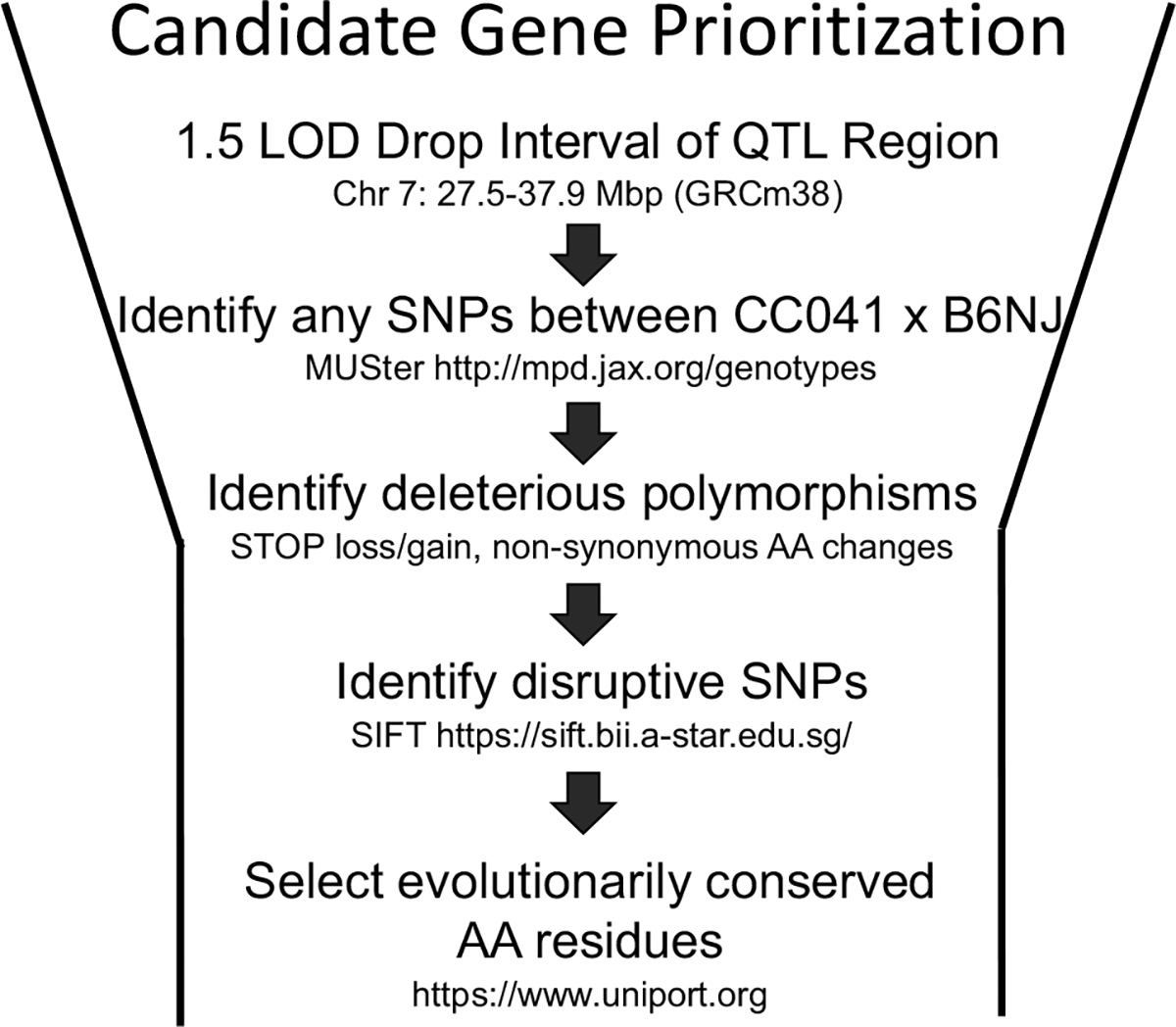
Strategy for identifying key candidate genes based on 1.5 LOD support interval and bioinformatic approaches to identify deleterious polymorphisms that are conserved and likely functionally relevant. Using the BioMart tool, we identified a list of protein-coding genes, lncRNA and miRNAs. We eliminated any that were in a region of IBD between B6J and the CC parental strain using the Mouse Phylogeny Viewer. We eliminated genes not expressed in the brain. We prioritized genes that had a nonsynonymous SNP between B6J and the CC parental strain, specifically targeting SNPs in coding sequence, splice regions, stop regions, frameshift and missense using MUSter (mpd.jax.org/genotypes) and that were likely to have a deleterious effect as determined by SIFT analysis (https://sift.bii.a-star.edu/sg/). We also prioritized genes with SNPs in evolutionarily conserved amino acid (AA) residues. Finally, we prioritized genes that met all three of the following criteria: previously identified with a phenotype of interest (i.e. brain structure/function or behavioral); expression differentially regulated by cocaine (using www.GeneWeaver.org); in a region that overlapped with previous QTL studies for initial cocaine-induced locomotion

The multi-step process outlined in Fig. 4 was used to identify potential candidate genes for cocaine locomotor response at the QTL regions on Chr 7 and Chr 11. The 10.4 Mb QTL region on Chr 7 contained 203 protein-coding genes. We identified 63,686 SN that differed between B6N and CC041. This included 283 different genomic features; 73 Gene Models, 5 miRNA, 29 *Scgb*-family members, 23 *Zfp* family members and 21 *RiK* or contig clones. There were zero stop loss or gain mutations and 152 missense mutations. Due to the one-to-many mappings (a SNP can be in multiple open reading frames), 168 mutations were determined to be tolerated, 21 were determined to be deleterious and 33 were unannotated (Table 4). Of the 21 deleterious mutations, only 3 (in *Ryr1*, *Ffar3* and *Lgi4*) were in protein residues that were conserved across species (mouse, human and at least one other mammal) suggesting that SNP had functional significance. We excluded *Nudt19*, *Nphs1* and *Cd22* genes as the protein residue at the location of the SNP is not conserved across species suggesting non-conservation of function. We also excluded the *Zfp* or *Scgb* family proteins due to the size of each gene family and their redundancy in the genome. A variant in *Wdr62* resulted in an A137T missense mutation in the WDR62 protein. However, while the reference strain, B6J has an A at that location, rat, human, pig and the NOD strain have a T.

The 25.1 Mb QTL region on Chr 11 contained 298,431 SNP differences between CC041and B6N. SNPs were in 140 protein coding genes (18 Rik, 33 GeneModel) and 3 miRNAs. 157,134 of these SNPs have registered rsID numbers. There were 658 coding non-synonymous substitutions (104 classified as missense variants), 281 synonymous substitutions, 268 lincRNA, 939 in 3’ or 5’ untranslated regions. Of the 658 coding SNPs, 642 were classified as tolerated and 16 (in 7 genes) were deleterious. SNPs in *Vrk2*, *Clhc1* and *Il9r* have a strong deleterious effect by SIFT analysis and are in a residue that is conserved across species. In addition, *Meis1* and *Psme4* are alternatively spliced in B6 vs CC041 and *Wdpcp* has an amino acid duplication in CC041 (rs230611425) that is not present in B6 and includes a conserved residue.

## Discussion

We employed F2 intercrosses of a traditional inbred mouse strain (B6N) to two CC strains (CC041 & CC004) to identify genetic loci associated with initial locomotor sensitivity to cocaine. The CC004 × B6N cross identified only one suggestive locus, but we were able to map three significant cocaine-induced locomotor response QTLs in the CC041 × B6N cross on Chrs 7, 11 and 14. The association between genotype and phenotype at the Chr 7 locus matches the mode of inheritance observed in the parental lines. At the Chr 11 QTL, however, we observed reduced locomotor response to cocaine that was associated with the B6N genotype, contrary to the parental phenotypes. All three CC041 × B6N QTL appear to be acting in an additive manner. However, there is also evidence for possible epistatic interactions based on allelic status at Chr 11 with both the Chr 7 and 14 loci, although the interactions did not reach significance. Using a multi-step bioinformatic approach, we identified potential candidate genes in the Chr 7 and Chr 11 QTL regions that could be mediating cocaine locomotor response. Below we compare our QTL regions to those identified in other mapping studies, discuss several high-priority candidate genes and share thoughts on how these data can be used in future studies aimed at expanding our knowledge about genetic and biological mechanisms that drive risk for CUD.

### Previous QTL studies for initial locomotor sensitivity to cocaine

Several previous studies have identified QTL for initial locomotor response to cocaine using panels of RI or congenic strains (Tolliver et al. 1994; Miner and Marley 1995; Phillips et al. 1998; Jones et al. 1999; Boyle and Gill 2001, 2009; Gill and Boyle 2003, 2008) and three have identified regions that overlap with our Chr 7 and 11 QTL regions. Gill and Boyle (2003) used congenic strains from the AcB/BcA panels derived by backcrossing the A/J inbred strain to B6J or the B6J strain to A/J and identified a QTL associated with initial cocaine locomotor response on Chr 7 for which increased cocaine response was driven by the B6J donor allele. Using a 25 Mb region around the peak at 51.7 Mb yields a region from 33.2 to 76.9 Mb which overlaps with the proximal end of our Chr 7 QTL interval. Tolliver et al. (1994) used C57BL/6 J × DBA/2 J (BXD) recombinant inbred strains to identify a locus on Chr 11 at 12.3 Mb where the B6J genotype was associated with higher cocaine response compared to DBA/2 J. Using a 25 Mb region around the peak yields a QTL region of 0 to 33.4 Mb which overlaps with our Chr 11 QTL interval. Finally, Jones et al. (1999) used BXD strains to identify a QTL interval on Chr 11 at 12.3 – 19.7 Mb at which the B6J genotype was associated with increased cocaine response. This QTL interval overlaps with our Chr 11 region as well as the locus identified by Tolliver et al (1994).

Kumar et al. (2013) identified a QTL peak on Chr 11 (1.5 LOD interval 35–57 Mb) that is associated with locomotor response to cocaine in an F2 intercross of B6 substrains, B6N and B6J. A causal variant in the *Cyfip2* gene was associated with low response to cocaine in B6N mice. The *Cyfip2* does not fall within our Chr 11 QTL LOD support interval, but we cannot rule out the possibility that *Cyfip2* could be contributing to the B6N-driven low cocaine response we observed.

CC041 mice are also deficient in acquiring cocaine self-administration (Schoenrock et al. 2020). Dickson et al. (Dickson et al. (2016)) identified QTL for cocaine self-administration in BXD mice on Chr 7 (30.4–30.6 Mb) within our QTL interval and on Chr 11 (46.18–50.57 Mb). The Chr 11 QTL is slightly outside our interval but does contain the *Cyfip2* gene. For both QTL, mice with the B6J genotype showed higher and more consistent cocaine infusions at lower doses of cocaine compared to animals with the DBA genotype.

### Potential candidate genes at the Chr 7 locus

Previous studies have used a variety of techniques to prioritize candidate genes including pre-existing knowledge about the function of genes in relation to the drug’s effects, the presence of parental strain polymorphisms and correlations with strain-specific gene expression changes. We used a systematic approach (Fig. 4) to prioritizing candidate genes based on the nature of identified SNPs. We prioritized the predicted impact of all SNPs, moving from most deleterious to least deleterious. For coding non-synonymous (missense) SNPs, we considered the resulting amino acid change and the conservation of the altered residue across species. Our approach resulted in the identification of three candidate genes within the Chr 7 interval, *Ryr1, Ffar3* and *Lgi4*. We briefly discuss evidence from the literature that lends support for each potential candidate. Further studies that consider the phenotypic consequences of disrupting gene function are necessary to demonstrate the veracity of each candidate.

The ryanodine receptor gene, *Ryr1*, has a SNP (rs47114500) that results in a G to A transition and a change from alanine to valine at residue 1118. The alanine at that residue is conserved in mouse, human and rabbit suggesting functional significance. Ryanodine receptors (*RyRs*) are a family of intracellular receptors responsible for Ca2 + release to support a number of intracellular actions (Chan et al. 2000; Chen et al. 2008, Grosskreutz et al. 2010; Zundorf and Reiser 2011; Forostyak et al. 2016; Kilpatrick et al. 2016; Egorova and Bezprozvanny 2019; Karagas and Venkatachalam 2019). Previous studies have identified an increase in *Ryr1* gene expression and elevation of RYR1 protein levels upon exposure to both methamphetamine and cocaine (Kurokawa et al. 2010; Kurokawa et al. 2011a, b) that is regulated by activation of dopamine D1 receptors (Kurokawa et al. 2010; Kurokawa et al. 2011a, b). Moreover, an induced mouse mutant containing a human *RYR1* polymorphism exhibits hypolocomotion (Zvaritch et al. 2009) suggesting that the *Ryr1* gene plays a role in locomotor behavior.

*Ffar3* has a SNP (rs46782561) between CC041 and B6NJ that changes a proline to a leucine at residue 192 and has been previously associated with cocaine-induced methylation (Feng et al. 2015). This specific residue has been predicted by AlphaFold (Jumper et al. 2021) to have hydrogen bonding with Thr 196 in its tertiary structure, contributing to the predicted detrimental effect of the variant. *Ffar3* encodes free fatty acid receptor 3, also known as GPR41, and is a type of G protein-coupled receptor essential for metabolism and immunity. (Kimura et al. 2020). This receptor has been shown to regulate the sympathetic nervous system by its expression on neurons involved in GBetaGamma-PLCbeta-MAPK signaling (Kimura et al. 2011).

The leucine-rich repeat LGI family, member 4 gene (*Lgi4*) has a histidine to tyrosine substitution at residue 531 that is predicted to have deleterious effects. The histidine at this residue is conserved across mouse, human and bovine LGI4 proteins. LGI proteins play a role in synaptic transmission and myelination in the nervous system (Kegel et al. 2013). In humans, mutations in LGI1 have been implicated in autosomal dominant lateral temporal lobe epilepsy (Nobile et al. 2009). A spontaneous mutation in the *Lgi4* gene (*Lgi4*^*clp*^) that results in loss of function was identified in the colony at the Jackson Laboratory (Henry et al. 1991). Studies using *Lgi4*^*clp*^ mice established the gene’s function in Schwann cell signaling in the peripheral nervous system (Bermingham et al. 2006). In situ hybridization studies characterized expression of *Lgi4* in the adult mouse medial septum, bed nucleus of the stria terminalis, substantia nigra and ventral tegmental area – brain regions that have been associated with addiction-relevant behaviors in mice (Herranz-Perez et al. 2010). A mouse knockout of *Lgi4* (*Lgi4*^*tm1.1(KOMP)Vlcg*^) is homozygous lethal but heterozygous female mice exhibit decreased locomotor activity in comparison to wildtype females suggesting a role for *Lgi4* in locomotor activity (https://mousephenotype.org).

Potential candidate genes at the Chr 11 locus controlling locomotor response to cocaine. We identified a transgressive QTL on Chr 11 in which reduced locomotor response to cocaine was driven by the B6N allele rather than the CC041 allele. Initially we found this result somewhat surprising given that CC041 mice show lower cocaine response than B6N mice (see Fig. 1d). We had crossed CC041 to B6N as B6N is genetically very similar to the B6J CC founder strain, and as such would not introduce an entirely new genetic background while still maintaining mapping power. However, investigation of the founder contribution in CC041 mice revealed that B6J contributed only 0.05% to the genetic background. Consequently, we introduced allelic combinations that would not have been present in the CC041 strain making the transgressive nature of the Chr 11 QTL less surprising. Although causal variants in the Chr 11 region are likely not responsible for the phenotypic effect observed in CC041, they are clearly contributing to low response to cocaine and warrant further consideration. Our bioinformatic analysis yielded SNPs in three high-priority candidate genes, *Clhc1, Il9r* and *Vrk2*. *Clhc1* and *Il9r* are expressed in the brain and have been implicated in neurobiological and behavioral phenotypes, but *Vrk2* has the strongest evidence as a candidate gene.

A *Vrk2* SNP (rs13466583) in CC041 mice results in a proline to leucine substitution at residue 319, an important structural residue predicted to have deleterious effects due to its predicted role in hydrogen bonding (Varadi et al. 2024). The vaccinia-related kinase (VRK) family of proteins are members of the kinome, the complete set of protein kinases encoded by the genome (Caenepeel et al. 2004). *Vrk2* is a serine/threonine kinase that regulates transcription factors in pathways ranging from apoptosis to tumor growth. It has been identified as a gene of interest 185 times across 92 human GWAS studies (Sollis et al. 2023). Of relevance to substance use disorders, *Vrk2* was a human GWAS hit for opioid and cannabis use disorders (Xu et al. 2023), was associated 18 times each with schizophrenia and neuroticism measures, seven times with alcohol consumption or alcohol use disorder (Karlsson Linner et al. 2019; Kranzler et al. 2019; Zhou et al. 2020; Saunders et al. 2022; Kember et al. 2023; Xu et al. 2023), and five times in smoking-related GWAS (Kichaev et al. 2019; Liu et al. 2019; Xu et al. 2020; Saunders et al. 2022; Xu et al. 2023). Variants in human VRK2 have also been associated with schizophrenia and major depressive disorder (Tesli et al. 2016) (Li and Yue 2018) (Yin et al. 2023). *Vrk2* KO mice also display increased depressive-like behaviors compared to wildtype littermates (Yin et al. 2023). Behavioral differences in these mice may be neurodevelopmental, as loss of *Vrk2* results in synaptic dysfunction and a reduction of dendritic spines in the ventral hippocampus (Yin et al. 2023).

We identified two deleterious polymorphisms in the coding sequence of the clathrin heavy chain linker domain containing 1 gene (*Clhc1*). A T to C transition changes a cysteine to arginine at residue 441 and an A to G transition changes threonine to alanine at residue 118. Polymorphisms in the human CLHC1 gene have been associated with bipolar disorder (Winham et al. 2014), major depressive disorder (Coleman et al. 2020) and brain structure differences (van der Meer et al. 2021). Behavior or brain structure phenotypes resulting from loss of function of *Clhc1* have not been reported in mice.

*Il9r* (interleukin 9 receptor) contains one SNP that differs between CC041 and B6N and results in a predicted deleterious coding-sequence substitution (S68L, S72L) in two of the five known *Il9r* transcripts. *Il9r* is differentially expressed in the nucleus accumbens and hippocampus of High Responder (bHR) and Low Responder (bLR) rats that were selectively bred for exploratory locomotor behavior in a novel environment (Clinton et al. 2011). *Il9r* knockout mice have extensive defects related to regulatory T-cell development (Elyaman et al. 2009) but no behavioral phenotypes have been observed.

We also identified transcript variants in *Meis1* (Meis homeobox 1) and *Psme4* (proteasome activator subunit 1) genes. *Meis1* is nearly ubiquitously expressed and functions as a transcription factor. Knocking out *Meis1* in mice results in embryonic lethality with prenatal alterations in the eye and vasculature and hematopoietic defects (Hisa et al. 2004). *Meis1* contains a frame-shift elongation mutation that differs between B6 (C) and NOD (CAAAAA) strains, resulting in a shorter transcript in B6 mice (ENSMUST00000118661.8). The gene is differentially expressed in the ventral tegmental area of C57BL/6 J mice given chronic cocaine vs. chronic saline (Campbell et al. 2021).

*Psme4* is ubiquitously expressed and differentially expressed in the nucleus accumbens and hippocampus of High Responder (bHR) and Low Responder (bLR) rats (Clinton et al. 2011). *PSME4* also showed differential expression in brain samples from patients with mood disorders (Sequeira et al. 2012). The SNP (rs235903318) that differs in B6 vs CC041 mice in transcript ENS-MUST00000154757.2 results in a frameshift that produces a truncated protein.

Finally, we identified an amino acid duplication in the WD repeat containing planar cell polarity effector gene (*Wdpcp*). The duplication is present in CC041 and not B6 and includes a residue that is conserved and has been implicated in alcohol consumption in human studies and the hypnotic effects of ethanol exposure in Drosophila (Liu et al. 2019; Saunders et al. 2022; O’Farrell et al. 2023).

We acknowledge that there may be transcriptional variants at QTL that contribute to behavioral variation. By focusing solely on coding variants, we risk overlooking important contributors, resulting in false negatives. Evaluating cis-acting expression QTLs (cis-eQTLs) within our LOD support intervals could help address this gap. However, the striatal eQTL data we previously collected in the Diversity Outbred population (Philip et al. 2023) reflect transcriptional variation in the B6J CC/DO founder strain, not the B6N substrain used in our current mapping study. Given potential differences in transcriptional variants between B6J and B6N, using this reference could lead to spurious associations and false positives.

The disparity in locomotor activity between the CC strains and B6N mice, even without cocaine administration, warrants further discussion. Our observation that CC041 and CC004 mice exhibit differences in locomotor activity relative to B6N across all three days of testing suggests that inherent differences in basal locomotor rank activity likely contribute to initial locomotor sensitivity to cocaine. In fact, we detected overlapping QTL regions across treatment days. The idea that some of our mapped QTL might reflect genetic differences in basal locomotor activity is not necessarily surprising. The regulation of both basal and psychostimulant-induced locomotor activity involves overlapping neural substrates and neurotransmitter pathways (Vezina and Kim 1999; Cornish and Kalivas 2001; Koob and Volkow 2016; Wu et al. 2020). These findings, together with our prior work demonstrating a link between cocaine-induced locomotor activity and cocaine’s rewarding and reinforcing effects (Schoenrock et al. 2020), support the premise that further investigation of candidate genes underlying our QTL regions will provide important insights into the genetic mechanisms underlying drug response and reward.

The availability of a rapidly evolving suite of bioinformatic resources that support systems genetics analyses of the CC and other commonly used inbred mouse strains has accelerated discovery of candidate genes and genetic variants that contribute to phenotypic variation. Our high-priority candidates can be further interrogated using multi-omic approaches to understand underlying genomic and transcriptomic mechanisms and cell-based assays to elucidate cellular and biological processes. Expanded behavioral testing of genetically engineered mice on appropriate genetic backgrounds can provide deeper insight into gene function. Our ultimate goal is to uncover biological mechanisms underlying CUD, with the aim of guiding the development of novel prevention and treatment strategies for patients. The specific genes and variants identified in our CC mapping study may not directly correspond to those in humans. Nonetheless, investigating these genes is likely to reveal key pathways and mechanisms that regulate the brain’s response to cocaine in humans and contribute to CUD risk in a genotype-dependent manner.

## Supplementary Material

Supplementary Material

**Supplementary Information** The online version contains supplementary material available at https://doi.org/10.1007/s00213-025-06901-z.

## Figures and Tables

**Table 1:** Number of mice phenotyped in CC004 and CC041 mapping populations

Population	Generation	Cross	F	M	Tot	Age in Days (StDev)

Low Responder (CC041)	F0	CC041/TauUnc (CC041)	12	12	24	66.2 (2.0)
		C57BL/6NJ (B6N)*	11	13	24	63.0 (2.8)
	F1	B6NxCC041 (BC)	13	13	26	61.7 (4.6)
		CC041xB6N (CB)	11	12	23	64.7 (3.4)
					**49**	
	F2	B6NxCC041 × B6NxCC041 (BCBC)	58	62	120	62.3 (3.0)
		CC041xB6N × CC041xB6N (CBCB)	59	53	112	62.3 (3.2)
		CC041xB6N × B6NxCC041 (CBBC)	66	63	129	62.0 (3.1)
		B6NxCC041 × CC041xB6N (BCCB)	37	50	87	63.1 (3.8)
					**448** **	
High Responder (CC004)	F0	CC004/TauUnc (CC004)	11	14	25	60.1 (2.8)
		C57BL/6NJ (B6N)*	11	13	24	63.0 (2.8)
	F1	B6NxCC004 (BC)	9	8	17	61.3 (2.3)
		CC004xB6N (CB)	9	10	19	62.5 (4.0)
					**36**	
	F2	B6NxCC004 × B6NxCC004 (BCBC)	38	39	77	61.4 (2.2)
		CC004xB6N × B6NxCC004 (CBBC)	38	39	77	65.0 (3.3)
		CC004xB6N × CC004xB6N (CBCB)	38	40	78	63.6 (3.1)
		B6NxCC004 × CC004xB6N (BCCB)	39	38	77	61.7 (2.9)
					**309** **	

* Same animals

** Represents number of mice phenotyped. Actual number genotyped was 444 (CC041) and 151 (CC004)

**Table 2:** QTL regions identified in the 3-day open field test

		Chr	Mb	Marker Name	LOD	*p*-value	1.5 LOD Interval (Mb)	Interval Size (Mb)	Decreasing Allele	CC Parent Haplotype

CC041 × B6N	Day 1 Distance	6	97.0	backupUNC060149691	6.29	0.001	51.5 – 111.4	59.8	CC041	129S1/SvImJ
		11	35.7	UNC111423133	3.88	0.074	18.0 – 70.8	52.8	B6N	NOD/ShiLtJ_WSB/EiJ
		14	84.6	JAX00385742	6.92	0.001	73.3 – 94.7	21.3	CC041	CAST/EiJ
	Day 2 Distance	6	37.1	backupUNC060073738	5.33	0.004	21.9 – 114.0	92.1	CC041	129S1/SvImJ
		7	28.9	UNC070790469	3.76	0.095	4.1 – 41.5	37.4	CC041	NOD/ShiLtJ_NZO/HlLtJ_NOD/ShiLtJ
		11	35.7	UNC111423133	5.00	0.008	21.7 – 51.6	29.9	B6N	NOD/ShiLtJ_WSB/EiJ
	Day 3 Distance	7	29.7	UNC070628845	6.24	0.001	27.5 – 37.9	10.4	CC041	NOD/ShiLtJ_NZO/HlLtJ
		11	30.9	backupJAX00025840	9.14	0.000	18.0 – 38.6	20.6	B6N	NOD/ShiLtJ_WSB/EiJ
		14	79.7	backupUNC140618860	4.43	0.022	12.6 – 97.2	84.5	CC041	NOD/ShiLtJ_129S1/SvImJ_CAST/EiJ
	Day 3- Day 2	7	29.7	UNC070628845	4.22	0.034	27.5 – 37.9	10.4	CC041	NOD/ShiLtJ_NZO/HlLtJ
	Distance	11	23.6	UNC110035818	6.48	0.001	12.6 – 37.7	25.1	B6N	NOD/ShiLtJ_WSB/EiJ
		14	79.7	backupUNC140618860	3.88	0.074	9.2 – 97.5	88.3	CC041	NOD/ShiLtJ_129S1/SvImJ_CAST/EiJ
CC004 × B6N	Day 3-Day 2 Distance	11	118.1	mUNC20502282	3.76	ns	4.9 – 118.5	113.6	B6N/CC041	A/J_129S1/SvImJ_NOD/ShiLtJ

All QTL regions above the suggestive genome-wide threshold (*p* = 0.1) using 1000 permutation for phenotypes in the 3-day open field test in the low-cocaine responding F2 population. *Chr* chromosome. *P*-values are derived from permutation analyses. Decreasing allele indicates the genotype associated with the lowest phenotype (CC041 = CC041/TauUnc; B6N = C57BL/6NJ). Collaborative Cross founder strain haplotype in QTL region is indicated in the last column. Presence of multiple founder strains indicate CC founder haplotype transitions in the QTL interval

**Table 3:** Two scan QTL results for CC041 × B6N F2 mapping population

		Full Model (allows for interaction)				Additive Model (no interaction allowed)		
		
	Chr(Pos1:Pos2)	Position 1 (Mb)	Position 2 (Mb)	LOD*_fv1_*	LOD*_i_*	Position 1 (Mb)	Position 2 (Mb)	LOD*_av1_*

	Threshold			**6.0**	**4.0**			**3.0**
Day 1 Distance	Chr6:Chr11	71.06	31.50	**6.4**	2.8	97.01	35.74	**3.6**
	Chr6:Chr14	71.06	84.63^+^	**8.0**	1.6	85.82	84.63^+^	**6.4**
	Chr11:Chr14	36.50^+^	84.63^+^	4.8	0.8	36.50^+^	84.63^+^	**4.0**
Day 2 Distance	Chr6:Chr7	105.06	39.67	4.3	0.6	37.09	28.90	**3.7**
	Chr6:Chr11	37.09^+^	35.74^+^	**6.4**	1.6	37.09^+^	35.74^+^	**4.8**
	Chr7:Chr11	28.90^+^	35.74^+^	4.7	0.4	28.90^+^	35.74^+^	**4.2**
Day 3 Distance	Chr7:Chr11	29.74^+^	20.98	**7.9**	1.0	29.74^+^	35.73	**6.9**
	Chr7:Chr14	29.74^+^	79.67^+^	5.5	0.4	29.74^+^	79.67^+^	**5.1**
	Chr11:Chr14	29.64	81.00	**6.6**	2.0	20.98	78.22	**4.6**
Day 3-Day 2 Distance	Chr7:Chr11	29.74^+^	20.98^+^	**6.1**	1.8	29.74^+^	20.98^+^	**4.3**
	Chr7:Chr14	36.76	26.48	4.7	0.3	29.74	79.67	**4.4**
	Chr11:Chr14	20.98^+^	72.60	5.8	1.7	20.98^+^	79.67	**4.0**

Gray shading indicates LOD scores that passed a lenient significance threshold. LOD*_fv1_* = comparing full model with QTL on the two chromosomes (ChrPos1:Pos2) to the single-QTL model, indicates evidence for a second QTL, allowing for the possibility of epistasis; LOD*_av1_* = comparing additive model with QTL on the two chromosomes (ChrPos1:Pos2) to the single-QTL model, indicates evidence for a second QTL, assuming no epistasis. LOD*_i_* = improvement in fit of full model over additive model (LOD*_fv1_*—LOD*_av1_*), indicates evidence for interaction

+ indicates same Chr position for full and additive model

**Table 4:** Number and class of genetic variants between CC041 and B6N at Chr 7 and Chr 11 QTL intervals

Variation Type	QTL Interval	
	
	Chr 7	Chr 11

3’ UTR	486	810
5’ UTR	151	129
Frameshift	0	2
In frame insertion	0	1
Missense	152	104
Non-coding exon	1152	1133
Spice acceptor	2	2
Splice donor	4	3
Splice region	79	99

## Data Availability

Parental strain and F1 data are provided within the manuscript or supplementary information files or are available upon request from the corresponding author. F2 phenotype and genotype data have been deposited in the public and freely available data repository, the QTL Archive at Mouse Phenome Database (phenome.jax.org;/centers/QTLA; Project IDs: Tarantino3 and Tarantino4).
