# Supplementary material for: Genetic mapping in collaborative cross mouse strains identifies loci that affect initial sensitivity to cocaine
Source: Psychopharmacology (Berl). 2025 Oct 27;243(6):1471–88. doi: 10.1007/s00213-025-06901-z (PMC12617008; doi:10.1007/s00213-025-06901-z)
Supplement: Supplementary file 1 — Supplementary file1 (DOCX 2026 KB) [file 213_2025_6901_MOESM1_ESM.docx]

**Supplemental Figure 1.** Individual chromosome plots for QTL for Day 3-Day 2 (**A**), Day 1 (**B**), Day 2 (**C**) and Day 3 (**D**) shown in Table 2. Lines depict genome-wide significance at *p* < 0.001 (red), *p* < 0.01 (green), *p* < 0.05 (blue) and *p* < 0.10 (black) as determined by permutation analyses.

**Supplemental Figure 2**. QTL map with all three days overlaid and depicted in red (Day 3, blue (Day 2) and black (Day 1). Lines depict genome-wide significance at *p* < 0.001 (red), *p* < 0.01 (green), *p* < 0.05 (blue) and *p* < 0.10 (black) as determined by permutation analyses.
